# Obesity increases cardiovascular mortality in patients with HFmrEF

**DOI:** 10.3389/fcvm.2022.967780

**Published:** 2022-09-08

**Authors:** Zhican Liu, Yiqun Peng, Wenjiao Zhao, Yunlong Zhu, Mingxin Wu, Haobo Huang, Ke Peng, Lingling Zhang, Sihao Chen, Xin Peng, Na Li, Hui Zhang, Yuying Zhou, Yongliang Chen, Sha Xiao, Jie Fan, Jianping Zeng

**Affiliations:** ^1^Department of Cardiology, Xiangtan Central Hospital, Xiangtan, China; ^2^Graduate Collaborative Training Base of Xiangtan Central Hospital, Hengyang Medical School, University of South China, Hengyang, China; ^3^Department of Scientific Research, Xiangtan Central Hospital, Xiangtan, China

**Keywords:** cardiovascular, mortality, body mass index, obese, heart failure, HFmrEF

## Abstract

**Background:**

High body mass index increases the risk of heart failure morbidity and mortality. It is unclear whether a high body mass index is associated with prognosis in patients with heart failure with mildly reduced left ventricular ejection fraction (HFmrEF). We retrospectively analyzed the effect of a high body mass index on the prognosis of patients with HFmrEF.

**Methods:**

We investigated the association between body mass index and cardiovascular death (death from any cardiovascular mechanism) in 1,691 HFmrEF patients (mean age, 68 years; 35% female) in Xiangtan Central Hospital. Using Cox proportional hazards models, body mass index was assessed as a continuous and a categorical variable.

**Results:**

Cardiovascular death occurred in 133 patients (82 males and 51 females) after 1 year of follow-up. After adjustment for established risk factors, there was a 7.5% increase in the risk of cardiovascular death for females for each increment of 1 in BMI. In contrast, changes in male body mass index were not significantly associated with cardiovascular death (*P* = 0.097). Obese subjects had a 1.8-fold increased risk of cardiovascular death compared with subjects with a normal body mass index. The hazard ratio for females was 2.163 (95% confidence interval: 1.150–4.066). Obesity was not significantly associated with cardiovascular death in males (*P* = 0.085).

**Conclusion:**

An increased body mass index is associated with an increased risk of cardiovascular death in patients with HFmrEF; however, this risk was mainly associated with female patients with HFmrEF and less with male patients with HFmrEF.

## Introduction

The global obesity epidemic is a significant public health concern (1). Approximately 39–49% of adults are overweight or obese (2). Cardiovascular disease (CVD) accounts for more than two-thirds of deaths due to a high body mass index (BMI) (3). Numerous studies have identified obesity as a significant risk factor for hypertension, CVD, and left ventricular hypertrophy. These are all important risk factors for heart failure (HF) (4, 5). HF is also a global epidemic that places enormous pressure on patients, caregivers, and the healthcare system (6, 7). Currently, patients with HF are classified as having reduced ejection fraction (HFrEF; LVEF ≤ 40%), mildly reduced ejection fraction (HFmrEF; LVEF 41–49%), or preserved ejection fraction (HFpEF; LVEF ≥50%) (8). Recent studies have shown that a higher BMI is more strongly associated with HFpEF risk than HFrEF (9), and since there are no studies on the association between BMI and patients with HFmrEF, it is unclear whether high BMI affects the prognosis of HFmrEF patients. Therefore, we retrospectively analyzed the effect of BMI on outcome events in patients with HFmrEF.

## Methods

### Body mass index

According to the World Health Organization (WHO) criteria, BMI is divided into underweight (BMI <18.5 kg/m^2^), normal weight (BMI 18.5–24.9 kg/m^2^), overweight (BMI 25.0–29.9 kg/m^2^), and obesity (BMI ≥ 30 kg/m^2^) (10). Therefore, we included patients with a BMI of 18.5–24.9 kg/m^2^ (normal weight) as the reference group based on accumulated epidemiological evidence (11).

### Participants

**Figure 1:**
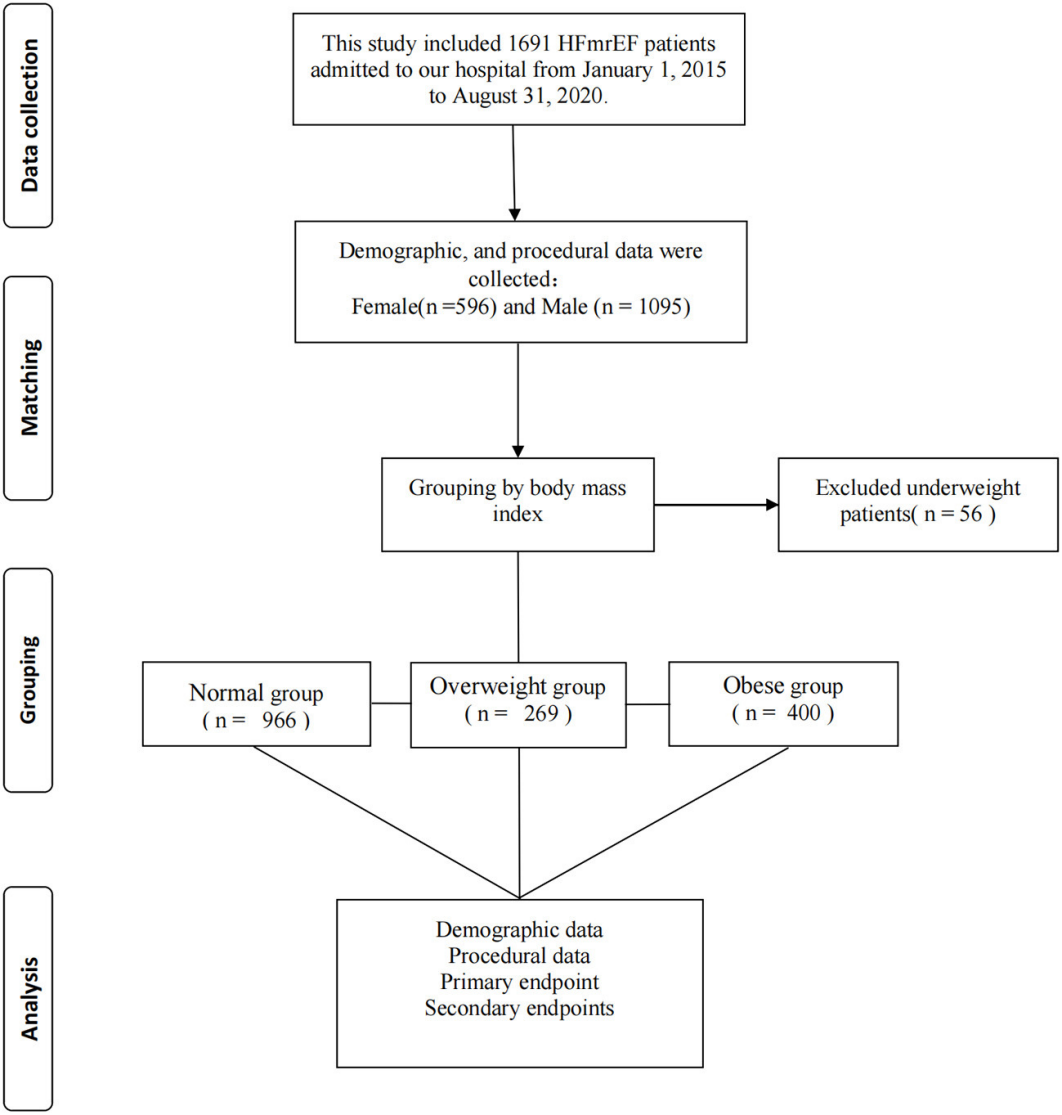
Flow diagram for participant screening, eligibility and analysis.

The study protocol was approved by the Ethics Committee of the Xiangtan Central Hospital (Xiangtan, China) and conformed to the principles outlined in the Declaration of Helsinki (12). Informed consent was obtained from all patients or their guardians before the study protocol was initiated. The requirement for written informed consent was waived because of the retrospective nature of the study, and only oral or telephonic consent was obtained. This study was based on the Outcome of Discharged HFmrEF Patients (OUDI-HF; ClinicalTrials.gov, number NCT05240118). The OUDI-HF study included 1,691 patients with HFmrEF who were admitted to our hospital between January 1, 2015, and August 31, 2020. Inclusion criteria included patients with HF with an LVEF of 41%−49% and a New York Heart Association HF score between II and IV. We are using Simpson's method to calculate the ejection fraction. The exclusion criteria were malignancies or other non-cardiac diseases with an expected survival of <1 year. After excluding 56 underweight patients, 966 were normal weight, 269 were overweight, and 400 were obese (Figure 1).

### Outcome

Demographic and procedural data were collected from hospital charts or databases. All study participants were followed-up on August 31, 2021. A panel of seven experienced physicians reviewed suspected cardiovascular events by examining the information obtained from hospital records and follow-ups, including clinical telephone interviews and community visits. In this investigation, the primary outcome of interest was cardiovascular death 1 year after discharge. *Cardiovascular death* was defined as “death from any cardiovascular mechanism, including acute myocardial infarction, sudden cardiac death, heart failure, stroke, cardiovascular surgery, cardiovascular hemorrhage, and other cardiovascular causes”.

### Statistical analysis

We used the Cox proportional hazards regression models stratified by cohort to examine the association of BMI and incidence of cardiovascular death. Sex-specific and sex-stratified analyses were performed. BMI was assessed as a continuous variable (increased risk was calculated for each increase of 1) and as a categorical variable. We adjusted for the following baseline covariates: age, smoking status, alcohol use, hypertension, hyperlipidemia, diabetes, coronary heart disease, atrial fibrillation, previous stroke, chronic obstructive pulmonary disease, renal insufficiency, New York Heart Association functional class, myocardial infarction, and percutaneous coronary intervention.

Clinical characteristics between the groups were compared using *t-*tests for continuous variables and chi-squared tests for categorical variables. The Kaplan–Meier method was used to estimate the incidence of cumulative events. *P*-values were obtained using the Kruskal–Wallis rank-sum test for continuous variables and the Fisher's exact probability test for count variables. The results were considered significant when the *P*-value was *P* < 0.05. All analyses were performed using R (http://www.R-project.org) and EmpowerStats (www.empowerstats.com, X & Y Solutions, Inc. Boston MA).

## Results

### Clinical characteristics

Approximately 17% of the males and 15% of the females were overweight. The prevalence of obesity was ~23% in males and 26% in females. Females had higher rates of hypertension and diabetes than did males (Table 1).

**Table 1:** Base-line characteristics according to the category of body-mass index.

**Characteristic**	**Men**			**Women**		
	**Normal**	**Overweight**	**Obese**	**Normal**	**Overweight**	**Obese**
	**(*N* = 631)**	**(*N* = 180)**	**(*N* = 248)**	**(*N* = 335)**	**(*N* = 89)**	**(*N* = 152)**
Age (year)	68.7 ± 12.0	66.1 ± 12.4	64.8 ± 13.5	69.8 ± 11.7	68.3 ± 11.6	69.5 ± 12.8
BMI(kg/m^2^)	22.7 ± 1.9	27.0 ± 1.2	31.0 ± 1.1	22.4 ± 1.9	26.8 ± 1.1	31.0 ± 1.1
Current smoker (%)	264 (41.8)	92 (51.1)	120 (48.4)	28 (8.4)	7 (7.9)	11 (7.2)
Current drinke (%)	71 (11.3)	24 (13.3)	41 (16.5)	4 (1.2)	2 (2.2)	2 (1.3)
Hypertension (%)	445 (70.5)	117 (65.0)	152 (61.3)	244 (72.8)	70 (78.7)	106 (69.7)
Hyperlipidemia (%)	109 (17.3)	33 (18.3)	69 (27.8)	74 (22.1)	17 (19.1)	40 (26.3)
Diabetes mellitus (%)	202 (32.0)	55 (30.6)	84 (33.9)	113 (33.7)	35 (39.3)	55 (36.2)
Coronary heart disease (%)	497 (78.8)	148 (82.2)	202 (81.5)	243 (72.5)	64 (71.9)	128 (84.2)
Atrial fibrillation (%)	123 (19.5)	25 (13.9)	38 (15.3)	66 (19.7)	23 (25.8)	16 (10.5)
Previous stroke (%)	84 (13.3)	17 (9.4)	29 (11.7)	53 (15.8)	5 (5.6)	15 (9.9)
COPD(%)	105 (16.6)	25 (13.9)	35 (14.1)	17 (5.1)	7 (7.9)	7 (4.6)
Renal insufficiency (%)	174 (27.6)	34 (18.9)	50 (20.2)	88 (26.3)	19 (21.3)	29 (19.1)
NYHA functional class [*n* (%)]						
II	261 (41.4)	101 (56.1)	112 (45.2)	131 (39.1)	37 (41.6)	45 (29.6)
III	243 (38.5)	55 (30.6)	91 (36.7)	123 (36.7)	33 (37.1)	62 (40.8)
IV	127 (20.1)	24 (13.3)	45 (18.1)	81 (24.2)	19 (21.3)	45 (29.6)
Myocardial infarction (%)	326 (51.7)	106 (58.9)	155 (62.5)	146 (43.6)	40 (44.9)	74 (48.7)
PCI (%)	213 (33.8)	66 (36.7)	115 (46.4)	88 (26.3)	23 (25.8)	43 (28.3)

The body-mass index was 18.5–24.9 in normal subjects, 25.0–29.9 in overweight subjects, and 30.0 or more in obese subjects. Values for continuous variables are given as means ± SD.

BMI, body mass index; COPD, chronic obstructive pulmoriary disease; NYHA, New York Heart Association; PCI, percutaneous coronary intervention.

### Body-mass index and the risk of cardiovascular death

**Figure 2:**
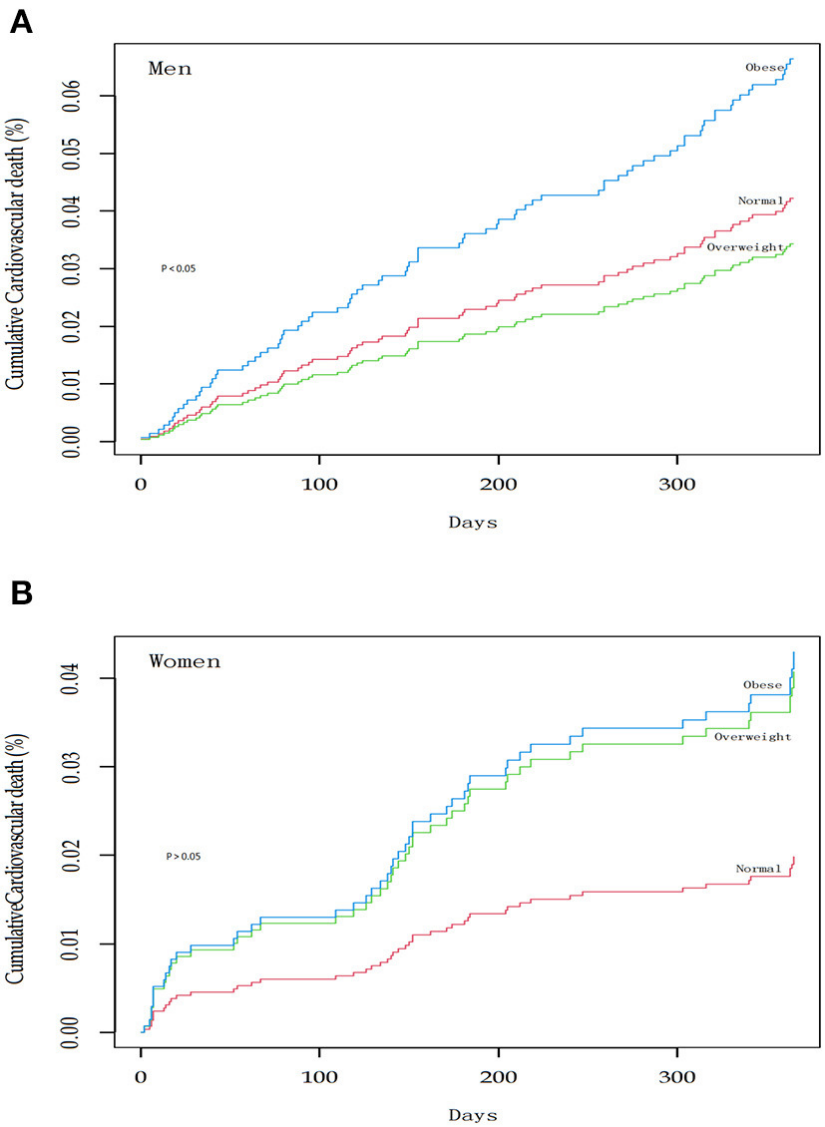
Cumulative incidence of 1-year cardiovascular death according to category of body-mass index at the base-line examination. The body-mass index was 18.5–24.9 in normal subjects, 25.0–29.9 in overweight subjects, and 30.0 or more in obese subjects. **(A)** One-year cumulative cardiovascular death in men. **(B)** One-year cumulative cardiovascular death in women.

After 1 year of follow-up, cardiovascular death occurred in 133 participants (82 males and 51 females). The crude cumulative incidence (Figure 2) and age-adjusted incidence rates (Table 2) of cardiovascular deaths increased across the female's BMI categories. In contrast, changes in male BMI were not significantly associated with cardiovascular death.

**Table 2:** Cumulative incidence of cardiovascular death among study participants according to the category of body-mass index at base line.

**Body-mass index**	**Men**		**Women**	
	**No. of death/no. of total patients**	**Age-adjusted 1-yr cumulative cardiovascular death**	**No. of death/no. of total patients**	**Age-adjusted 1-yr cumulative cardiovascular death**
		**Hazard ratio (95% CI)/*P*-value**		**Hazard ratio (95% CI)/*P*-value**
Normal (18.5–24.9)	51/631	1	22/335	1
Overweight (25.0–29.9)	9/180	0.706 (0.347, 1.436)/0.336	9/89	1.864 (0.855, 4.063)/0.117
Obese (≫30.0)	22/248	1.283 (0.777, 2.121)/0.330	20/152	2.087 (1.139, 3.825)/0.017

CI denotes confidence interval. ≫ Means greater than or equal to.

After adjusting for age, an increase of 1 in BMI was associated with a 4.8% increase in the overall risk of cardiovascular death [hazard ratio (HR) 1.048; 95% confidence interval (95% CI) 1.004–1.095; *P* = 0.033]. Among them, the risk of cardiovascular death was increased by 7.1% in females (HR 1.071; 95% CI 1.002–1.144; *P* = 0.042), and the association between changes in BMI and cardiovascular death in males was not significant (HR 1.029; 95% CI 0.971–1.090; *P* = 0.334) (Table 3, Model IA). There was no difference in the odds of cardiovascular death between overweight subjects and subjects with a normal BMI (*P* > 0.05). Obesity had a 57% increased risk of cardiovascular death (HR 1.570; 95% CI 1.073–2.298; *P* = 0.020), including a 108.7% increase in females (HR 2.087; 95% CI 1.139–3.825; *P* = 0.017), and males were not associated with cardiovascular death (HR, 1.283; 95% CI 0.777–2.121; *P* = 0.330) (Table 3, Model IB).

**Table 3:** Results of multivariable cox proportional-hazards models examining the relation of body-mass index to the risk of heart failure.

**Model and category of body-mass index**	**Sex-specific analyses**				**Sex-stratified analyses**	
	**Men (*****N*** = **1,059)**		**Women (*****N*** = **576)**		**Total (*****N*** = **1,635)**	
	**Hazard ratio**	***P*-value**	**Hazard ratio**	***P*-value**	**Hazard ratio**	***P*-value**
	**(95% CI)**		**(95% CI)**		**(95% CI)**	
I. Age adjusted:1-year cardiovascular death						
A. Body-mass index as a continuous variable (per increment of 1)	1.029 (0.971, 1.090)	0.334	1.071 (1.002, 1.144)	0.042	1.048 (1.004, 1.095)	0.033
B. Body-mass index as a categorical variable						
Normal (18.5–24.9)	1		1		1	
Overweight (25.0–29.9)	0.706 (0.347, 1.436)	0.336	1.864 (0.855, 4.063)	0.117	1.032 (0.615, 1.732)	0.904
Obese (≫30.0)	1.283 (0.777, 2.121)	0.330	2.087 (1.139, 3.825)	0.017	1.570 (1.073, 2.298)	0.020
II. Full adjustment:1-year cardiovascular death						
A. Body-mass index as a continuous variable (per increment of 1)	1.052 (0.991, 1.117)	0.097	1.076 (1.004, 1.154)	0.039	1.065 (1.018, 1.115)	0.006
B. Body-mass index as a categorical variable						
Normal (18.5–24.9)	1		1		1	
Overweight (25.0–29.9)	0.812 (0.395, 1.669)	0.572	2.048 (0.919, 4.564)	0.080	1.178 (0.698, 1.990)	0.539
Obese (≫30.0)	1.573 (0.939, 2.635)	0.085	2.163 (1.150, 4.066)	0.017	1.815 (1.228, 2.684)	0.003

The normal body-mass index category was the reference category. CI denotes confidence interval. ≫ Means greater than or equal to.

Full adjustment for age, current smoker; current drinker; hypertension; hyperlipidemia; diabetes mellitus; coronary heart disease; atrial fibrillation; Previous stroke; chronic obstructive pulmoriary disease; renal insufficiency; New York Heart Association functional class; myocardial infarction; percutaneous coronary intervention.

**Figure 3:**
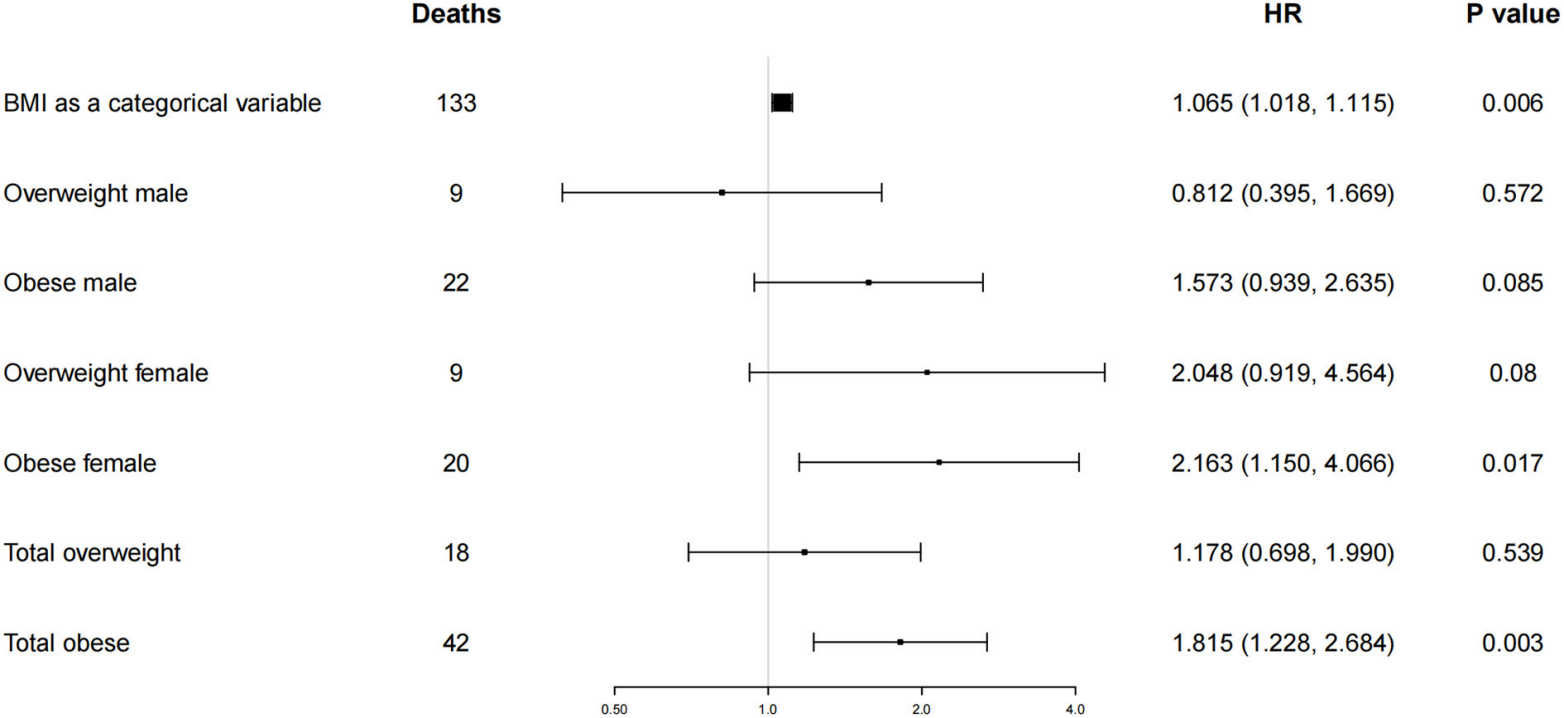
Risk of cardiovascular death in overweight and obese subjects according to body mass index category at baseline examination. I bars represent the 95 percent confidence intervals for the hazard ratios. Hazard ratios were adjusted for age, smoking status, alcohol use, hypertension, hyperlipidemia, diabetes, coronary heart disease, atrial fibrillation, previous stroke, chronic obstructive pulmonary disease, renal insufficiency, New York Heart Association functional class, myocardial infarction, and percutaneous coronary intervention at base line. Normal weight (body-mass index,18.5 to 24.9) was the reference category.

After adjusting for all covariates, a BMI increase of 1 was associated with a 6.5% increase in the overall risk of cardiovascular death (HR 1.065; 95% CI 1.018–1.115; *P* = 0.006). Among them, the risk of cardiovascular death was increased by 7.6% in females (HR 1.076; 95% CI 1.004–1.154; *P* = 0.039), and the change in BMI was not significantly associated with cardiovascular death in males (HR 1.052; 95% CI 0.991–1.117; *P* = 0.097) (Figure 3 and Table 3, Model IIA). There was no difference in the odds of cardiovascular death among overweight subjects compared to those with a normal BMI (*P* > 0.05). The risk of cardiovascular death was increased by 81.5% in obese individuals (HR 1.815; 95% CI 1.228–2.684; *P* = 0.003), including 116.3% in females (HR 2.163; 95% CI 1.150–4.066; *P* = 0.017), whereas males were not associated with cardiovascular death (HR 1.573; 95% CI 0.939–2.635; *P* = 0.085) (Figure 3 and Table 3, Model IIB).

## Discussion

This study found that high BMI increased 1 year cardiovascular mortality in female patients with HFmrEF after adjusting for covariates. However, increased BMI was not associated with 1-year cardiovascular mortality in male HFmrEF patients.

Recent studies have shown that a higher BMI is more strongly associated with HFpEF risk than HFrEF (9), and other studies have confirmed this view (13). Participants with overweight and grade 1 obesity (BMI: 30.0–34.9 kg/m^2^) had a 38% and 56% higher risk of HFpEF (13), respectively. However, there are also different findings called the obesity paradox. These studies suggest that overweight or grade 1 obese patients have better clinical outcomes than normal weight patients with HF, which is more common in patients with HFrEF than in those with HFpEF (4, 5, 14). This may be related to lower epicardial adipose tissue in normal-weight HF patients, which increases the risk of death in HF patients (15–17). However, there are also different views. The Metabolic Exercise test data combined with Cardiac and Kidney Indexes (MECKI) Score Research Group found that the obesity paradox in HFrEF patients may be related to patient selection bias (18). This paradox may disappear after considering exercise capacity and other cardiopulmonary exercise test variables. The studies mentioned above, as well as ours, have demonstrated a correlation between HF and BMI. Nevertheless, the difference is that other studies have failed to explain the relationship between HFmrEF and BMI, while our study focused on the effect of BMI on the prognosis of patients with HFmrEF.

Despite the obesity paradox, weight loss is still recommended in patients with HF (19–23). Obesity is not only associated with diseases of the cardiovascular system, but also with diseases such as stroke, venous thromboembolic disease, and pulmonary hypertension (24, 25). Numerous studies have identified obesity as a significant risk factor for hypertension, CVD, and left ventricular hypertrophy. However, hypertension, CVD, and left ventricular hypertrophy are also important risk factors for HF development (4, 5). The Framingham Heart Study found that, with increasing BMI, the incidence of HF increased in both males and females (26). Several studies have confirmed this finding (15, 27). Studies on bariatric surgery have shown improved left ventricular systolic function in patients with post-operative HF (28, 29) and a reduction in HF hospitalizations (30). At the same time, the HF guidelines also consider a high risk associated with severe obesity (8).

### Limitations

This study had several limitations. First, this was a retrospective study to minimize bias in patient selection; however, unobserved confounders remained. Second, our study exclusively recruited patients from China from an isolated population at a local heart center, thereby lacking the diversity to justify the uniformity of the findings. Lastly, data from patients undergoing long-term treatment for heart disease with statins, renin/angiotensin blockers, and beta-blockers were not included in the protocol for this study. We were unable to assess the effects of these drugs or their effects on the long-term morbidity and mortality of the enrolled subjects.

## Conclusion

The present study found that obesity increased the1year risk of cardiovascular death in females with HFmrEF (per an increase of 1 in the BMI in female patients, the risk of death increased by 7.6%, and the risk of death in obese patients was twice that of normal-weight patients), but not in males. Further research is warranted to understand complex sex-related risk differences among patients with HFmrEF. A better understanding of sex-specific risk factors may help in developing strategies to improve outcomes for this critical disease.

## Data availability statement

The original contributions presented in the study are included in the article/Supplementary material, further inquiries can be directed to the corresponding author.

## Ethics statement

The studies involving human participants were reviewed and approved by Xiangtan Central Hospital (No.20211036). Written informed consent for participation was not required for this study in accordance with the national legislation and the institutional requirements.

## Author contributions

ZL, YP, and WZ established the idea to study the heart failure with mildly reduced ejection fraction in Chinese population, and helped in writing main ideas for this research, main results and discussion of the findings. ZL was a major contributor in writing the manuscript. YZhu, MW, HH, KP, LZ, and JF interpreted statistical analysis and conducted multivariate analysis to prove the main findings of this project. SC, XP, NL, HZ, YZho, YC, and SX collected data and followed-up. JZ contributed on editing this manuscript and giving advice for the main authors to organize the manuscript and ideas of the project. All authors contributed to the article and approved the submitted version.

## Funding

This study was supported by Scientific Bureau of Xiangtan City (SF-YB20201023), Xiangtan City, Hunan Province, China and Committee of Development and Reform of Hunan Province (2019-875), Changsha, Hunan Province, China. The funders had no roles in study design, data collection and analysis, decision to publish or preparation of the manuscript.

## Conflict of interest

The authors declare that the research was conducted in the absence of any commercial or financial relationships that could be construed as a potential conflict of interest.

## Publisher's note

All claims expressed in this article are solely those of the authors and do not necessarily represent those of their affiliated organizations, or those of the publisher, the editors and the reviewers. Any product that may be evaluated in this article, or claim that may be made by its manufacturer, is not guaranteed or endorsed by the publisher.
